# Trends of diarrhoeal diseases in children under five years in the War Memorial Hospital-Navrongo, Ghana: 2010-2013

**DOI:** 10.11604/pamj.supp.2016.25.1.6173

**Published:** 2016-10-01

**Authors:** Maria Anyorikeya, Donne Kofi Ameme, Kofi Mensah Nyarko, Samuel Oko Sackey, Edwin Afari

**Affiliations:** 1 Department of Epidemiology and Diseases Control, School of Public Health, University of Ghana; 2 Ghana Field Epidemiology and Laboratory Training Program, School of Public Health, University of Ghana

**Keywords:** Diarrhoea, children under five years, seasonal, distribution, Ghana

## Abstract

**Introduction:**

Diarrhoea is the third leading cause of hospital morbidity in children under five years in the War Memorial Hospital (WMH). With the current changes in climate, little is known about the seasonal and spatial distribution of diarrhoeal diseases in the WMH. We determined trends of diarrhoeal diseases in children under five years in the WMH.

**Methods:**

We reviewed secondary data of children under five years who attended the WMH and were diagnosed of diarrhoea. Diarrhoea was defined as a clinician's diagnosis of the passage of three or more watery stools a day in a child under five years in the WMH. Descriptive data analysis was done and expressed as frequencies and relative frequencies. Monthly proportions of diarrhoea and rainfall figures were presented to show seasonal distributions of cases. Geographical distribution of cases was determined using Epi Info and Arc GIS.

**Results:**

A total of 865 diarrhoeal cases in children under five years reported to the hospital. Out of this, 425 (49.13%) were female children with 346 (40%) aged 0-11 months. The highest peak occurred in the rainy season from May to August. However, there was a weak negative relationship between diarrhoeal diseases and rainfall for the whole study period. Cases were clustered in the northeastern part of the Kassena Nankana Municipality (KNM).

**Conclusion:**

The most affected age group was in 0-11months. Majority of cases were from the North Eastern part of the Municipality. There was seasonal variation of diarrhoeal diseases. Diarrhoeal diseases have the highest occurrence in the rainy season but an increase in rainfall does not necessarily lead to an increase in diarrhoeal cases. Intervention to reduce diarrhoea should be intensified before the rainy season and in the northeastern parts of the municipality.

## Introduction

Diarrhoea is an important cause of morbidity and mortality among children aged five years and below. Diarrhoea is the second cause of childhood mortality causing about two billion deaths annually [1]. About 80% of these deaths occur in developing countries [1, 2]. In sub-Saharan Africa, the incidence of diarrhoea has been linked with poverty as poor communities lack adequate sanitation and water supply [3]. Seasonality of diarrhoeal diseases have been reported by different authors worldwide [2, 4]. In Ghana, rotavirus was more prevalent in the dry season than the rainy season in the Kassena Nankana District [5]. Communities with limited access to social amenities and those closer to health facilities have been reported to have higher incidence of diarrhoea as those in settings closer to health facilities may be more likely to report to health facilities than setting further away [5, 6]. The prevalence of diarrhoea in Ghana is high. It was established that by 2006, diarrhoea was taking the lives of over 14,000 Ghanaian children under age five annually; and it was second to malaria as a leading cause of death in children [7]. For the year 2011, 113,786 cases of diarrhoea were recorded in Ghanaian children under five years. About 2,318 of these cases had severe dehydration and 354 died giving a case fatality rate of 0.31% [8]. The highest incidence rate of 3,611.2/100,000 population was recorded in the Upper East Region of Ghana where Kasenna Nankana Municipality (KNM) is located in 2011. In the KNM, diarrhoeal diseases accounted for 17% of all diseases recorded in 2009 and remain the third leading cause of hospital admissions in the War Memorial Hospital (WMH). Studies on trends of diarrheal diseases have been done in the KNM on acute watery diarrhoea especially rotavirus related diarrhea but not much has been done on all cause diarrhoeal diseases in children under five years. With the recent changes in climate, increases in heat waves and floods there may be changes in the distribution of diarrhoeal diseases over time. We analyzed routine surveillance data to assess trends of diarrhoeal diseases in children under five years reporting to the WMH from 2010-2013.

## Methods

**Study design and study site:** a retrospective analysis of routine diarrhoeal surveillance data records of children under five years of age who attended the WMH from 2010-2013 was done. We defined diarrhoea as a clinician's diagnosis of the passage of three or more watery stools a day in a child under five years in the WMH from 2010 to 2013. The study was conducted in the WMH which is located in KNM but also serve Kassena Nankana West District (KNWD) as it is the only municipal hospital in the area.

**Sampling method and data collection:** all hospital records of diarrhoeal diseases in children under five years who sought health care at the WMH from 2010 to 2013 were reviewed using a data capture form. We collected demographic characteristics such as age, sex and residential address of diarrhoeal cases of children under five years from consulting room registers. We obtained summary sheets of monthly OPD attendance of all cause morbidity in children under five years in the WMH by their demographic characteristics. We acquired meteorological data on monthly rainfall from 2010 to 2013 from the meteorological agency of the KNM. Arc files of the map of the KNM and coordinates of the communities were collected from the Upper East Regional Health Directorate.

**Data analysis:** we conducted descriptive statistical analysis of the data by time, place and person. Age was aggregated into two categories; 0-11 months and 12-59 months. This categorization of age groups was to highlight the critical age group of 0-11 month old, among whom diarrhoeal diseases are most severe. The total number of children under five years who sought health care in the hospital from 2010-2013 was grouped according to the various months they were seen at the hospital. We calculated median and inter quartile ranges for continuous variables and calculated frequencies and relative frequencies for categorical variables. An Autoregressive Integrated Moving Average (ARIMA) analysis was performed to find out the relationship between rainfall and diarrhoeal diseases. A cross correlation was also performed at different months to find out the relationship between rainfall and the time (months) it takes for the occurrence of diarrhoeal diseases. We considered lags (delays in effect) up to 3 months.

**Geographical distribution of diarrhoeal diseases:** we run frequencies of diarrhoeal diseases by patient residential address for each year using Epi Info version 7. We saved the frequencies as a Microsoft Access file and exported into Arc Map Version 10.2. The arc file for the KNWD and the KNM was carved out of the regional map. Maps showing the distribution of diarrhoeal diseases that reported to the WMH in the study period were drawn on the arc file in such a way that only selected districts with diarrhoeal diseases were shown on the map.

**Ethical considerations:** ethical clearance was obtained from the Ghana Health Service Ethics Review Committee and administrative authorization was obtained from the medical superintendent of the WMH. Names of patients were not included in data collection and analysis.

## Results

A total of 865 diarrhoeal cases in children under five years were recorded for the period 2010 to 2013. Of these 346.0 (40%) were between the ages of 0-11 months and 425 (49.13%) were females. The median age of the patients was 12 months (interquartile range: 7- 24). The incidence of diarrhoeal diseases recorded in the hospital for the study period was 20/1000. Of the records reviewed, 30.0 (3.47%) had no sex recorded (Table 1). Majority of the diarrhoeal diseases 745 (86.13) recorded in the hospital were from the KNM. Clinically, majority of the diarrhoea cases 655 (75.7%) were non-bloody (non-dysentery) whilst 210 (24.3%) were classified as bloody diarrhoea (dysentery).

**Table 1:** Proportions of diarrhoeal diseases in children under five years by background characteristics in WMH: 2010-2013

Characteristics	Year				
	2010	2011	2012	2013	Total
	n(%)	n(%)	n(%)	n(%)	
**Age (months)**					
0-11	104(48.60)	96(41.60)346(40.00)	58(33.00)	88(36.00)	346(40.00)
12-59	110(51.40)	135(58.40)	120(67.00)	154(64.00)	519(60.00)
**Sex**					
Female	94(43.93)	115(49.78)	89(50.00)	127(52.50)	425(49.13)
Male	105(49.07)	109(47.19)	81(45.51)	115(47.50)	410(47.40)
Missing	15(07.00)	7(3.03)	8(4.49)	0(0.00)	30(3.47)
**Geographic Location**					
KNM	198(92.50)	197(85.30)	148(83.15)	203(83.90)	745(86.13)
KNWD	16(7.50)	34(14.70)	30(16.85)	39(16.10)	120(13.87)
**Total**	**214(100.00)**	**231(100.00)**	**178(100.00)**	**242(100.00)**	**865(100.00)**

### Seasonal variations in occurrence of diarrhoeal diseases

**Figure 1:**
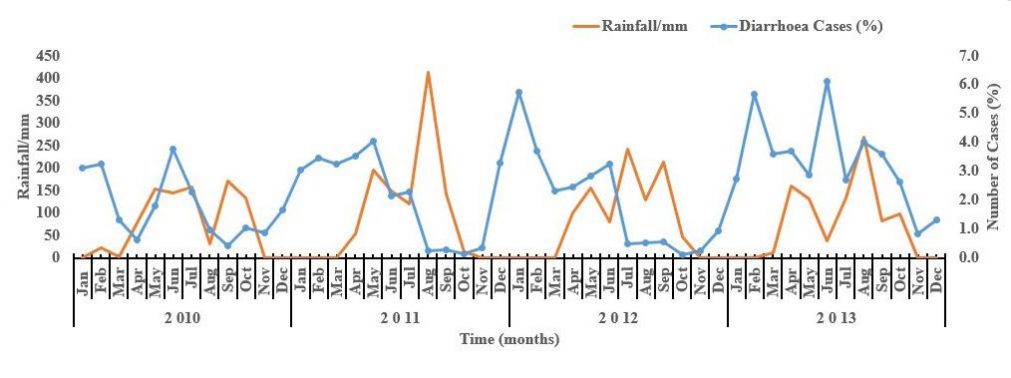
Seasonal variations in proportions of diarrhoeal diseases in children under five years by rainfall/mm in War Memorial Hospital

Over the study period, seasonality of diarrhoeal diseases can be described as erratic and irregular and each year shows a different picture. Despite this, diarrhoeal diseases showed a bimodal distribution each year; a high and low peak. The highest peaks occurred in June in 2010, May in 2011, January in 2012 and June in 2013. The minor peaks occurred in February in 2010, December in 2011, June in 2012 and February 2013. The highest rainfall for the study period was observed between August and September 2011. This was followed by an increase in diarrheal diseases from November 2011 to January 2012. Each year however, from October to March of the following year, there were no rains. At the same time, high proportions of diarrheal disease were observed in the hospital. The highest proportion of diarrhoea was observed in June, 2013 with a corresponding decrease in rainfall (Figure 1).

### ARIMA analysis

To better understand whether there was a statistically significant relationship between rainfall and diarrhoeal diseases, an ARIMA regression was run. This showed weak negative relationships between cases and rainfall from January 2010 to December 2013 overall, and for each year in the study except 2013 which showed a weak positive relationship as shown in the p-values and coefficient values. Thus from January 2010 to December 2013; -0.03 P>0.05, 2010; -0.03 P>0.05, 2011; 0.004 P>0.05. For 2012; 0.007 P >0.05, and 2013; 0.002 P>0.05. This may account for the erratic pattern in cases and rainfall figures as shown in the graph. A cross correlation of diarrhoea cases and rainfall with different lags showed a weak negative correlation of rainfall with cases when compared for the same month (lag 0) but increases when lagged up to 3 months suggesting rainfall is correlated with a decrease in cases up to 3 months backwards.

### Geographic distribution of diarrhoeal diseases

**Figure 2:**
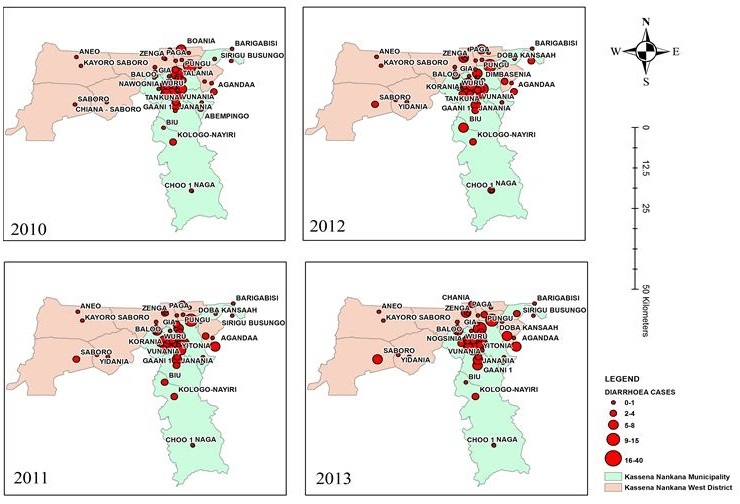
Geographical distribution of the diarrhoeal diseases in children under five years reporting to War Memorial Hospital, 2010-2013

Out of the 865 cases of diarrhoeal diseases recorded, 120 (13.87%) cases were from the KNWD and 745 (86.13) were from the KNM. A total of 57 communities from both KNM and KNWD had children reporting to the WMH with diarrhoeal diseases. Throughout the study period, the highest number of diarrhoeal diseases among children under five years recorded in the hospital 170 (21.66%) was from the Nogsenia community in the KNM, where the hospital is located. Doba community recorded the second highest number of diarrhoeal diseases 65 (8.28%). Generally over the four years under study, there was no change in the trend in the geographical distribution of diarrhoeal diseases recorded in the WMH (Figure 2). In all four years, most cases were clustered around the north eastern parts of the district where the WMH is located. Few cases were scattered in the KNWD. Upper Nangalgenia, Nogsenia, Doba and Pungu communities recorded diarrhoeal diseases between 16-40 cases. Gongnia recorded diarrhoeal diseases between 15-10 cases. The rest of the communities recorded diarrhoeal diseases between 1-9 cases each year throughout the study period.

## Discussion

In our study, diarrhoeal diseases among children under five years old occurred throughout the year. The study found that the most affected age group of children with diarrhoeal diseases was between 0-11 months old. This is consistent with works conducted by many researchers [5, 9].

Our findings on seasonality of diarrhoeal diseases occurring in the rainy season is similar to trends reported by other researchers Kelly-Hope et al. These researchers observed that seasonal trends contributed to diarrhoeal diseases in children under five years of age in Vietnam [10]. The most likely explanation for this could be the fact that poor sanitary conditions are prevalent during the rainy season. Rainy seasons in these areas are characterized by a lot of flies, which serve as vectors for transmitting diarrhoeal diseases. In addition, the rains wash fecal matter from human open defecation and from animals' feacal waste into water bodies, which serve as sources of drinking water for humans, particularly in the KNM. Similarly, a study in Burkina Faso, identified human contamination of water bodies which served as sources of drinking water for humans as a potential risk factor for diarrhoea caused by rotavirus [9]. Our findings on the occurrence of two peaks of diarrhoea each year supports the evidence of a study conducted in Bangladesh that all diarrhoea excluding cholera showed a bimodal seasonality with the first peak occurring prior to the high rainfall period and the second peak occurring towards the end of the high rainfall [4]. In our study, the minor peaks occurred between December and February before the rainy season (May to August) with the exception of 2012 where it occurred in June. The major peaks however occurred in the raining season with the exception of 2012 where it occurred in January. Similarly, previous studies in the then KND observed that diarrhoea due to rotavirus was more prevalent in the dry season whiles diarrhoea due to other pathogens was prevalent in the rainy season [5]. This could be a possible reason for the occurrence of high proportions of diarrhoeal diseases in between December and February each year (dry season). Diarrhoea recorded in this group may be due to rotavirus.

Rainfall patterns over the four years did not follow a regular trend as high peaks occurred in different months over the four years. The ARIMA analysis showed a weak negative relationship between diarrhoeal diseases and rainfall for the whole study period. Similarly, in a study on the seasonal effects of reported cases of diarrhoea, rainfall did not appear to have a direct effect on acute diarrhea [11]. In another study to estimate the effects of rainfall and temperature on the number of non-cholera diarrhoea cases it was found that the number of cases of diarrhoea increased by 3.9% for every 10mm decrease below a threshold of rainfall and increased by 5.1% for every 10mm increase in rainfall above the threshold. These researchers concluded that the number of non-cholera dirrhoea increases with both an increase or decrease in rainfall above a threshold [4]. The location of the hospital in the Nogsenia community in the KNM gives easier geographical accessibility to inhabitants of the municipality. This accounts for the highest record of diarrhoeal diseases from this community in the municipality. Distance of place of residence from the health facilities has been observed to influence reporting of diarrhoeal diseases to health facilities [5]. In addition, the provision of better health service as the WMH is the only Hospital in the KNM will attract parents of children under five years with diarrhoeal diseases.

Another reason for this could be the presence of community based health facilities such as the Community Based Health Planning and Services (CHPS) compounds dotted around the KNWD, which serves as the first point of call for its inhabitants. Children under five years living in areas further away from the WMH who have diarrhoea are likely to visit these CHPS facilities and therefore will not form part of the data analyzed at the WMH. The North Eastern parts of the Municipality have the highest number of children under five years (about 13,498) [12]. It is therefore not surprising that it constituted the highest proportions of diarrhoeal diseases. This part of the Municipality has the greatest population of individuals as a result, there is overcrowding leading to poor sanitation and risk of diarrhoeal disease. Contrary to the findings of our study, Chaikaew et.al, studied spatial distribution and hot spot of diarrhoea in Thailand, and found that hotspots had shifted from urban villages to highland villages, which had limited access to portable water, infrastructure, and health systems. This difference in results may be because their study was community based and ours was a hospital-based study and therefore was limited by the fact that it did not cover the whole Municipals' population.

Furthermore, clustering of diarrhoeal diseases within the North Eastern parts of KNM from 2010 through 2013 may also underscore the health seeking behavior of the people. It is likely that the urban dwellers in the North Eastern parts of KNM are more likely to seek modern health care for diarrhoea as compared to the rural inhabitant of the KNM and the KNWD. This could be linked with high socio-economic status and educational levels of caregivers of children under five years in the North Eastern parts of the Municipality as compared to the other communities in the KNM and the KNWD as found in other studies [3, 12]. In these two studies families with higher socio economic status had a greater chance of seeking orthodox health care for ailments including diarrhoea.

### Limitations

The definition of diarrhoeal diseases was based on a clinician's diagnosis as a result, there might be misclassification of diarrhoea cases if a clinician wrongly diagnosed a child under five years as having diarrhoea. The study only assessed trends of diarrhoeal diseases in the WMH but did not assesse other health facilities in the community as a result, findings from this study cannot be generalized for the whole community.

## Conclusion

There was a seasonal trend in the occurrence of diarrhoeal diseases reported in children under five years in the WMH. Majority of diarrhoeal cases occurred in the rainy season from May to August with lower levels recorded mainly in the dry seasons. Though the diarrhoeal cases in children under five years were from a wide range of communities, there was clustering of the cases in communities around the North Eastern parts of the KNM. We suggest that interventions targeted at reducing diarrhoeal diseases should be enforced before the rainy seasons and in the north eastern parts of the municipality.
